# An approach of dual attention-driven multi-levelled graph neural architecture for rich-structural social graph analysis and representation learning

**DOI:** 10.1371/journal.pone.0351922

**Published:** 2026-06-26

**Authors:** Linh Nguyen Thi My, Tham Vo

**Affiliations:** 1 Faculty of Information Technology, Van Lang School of Technology, Van Lang University, Ho Chi Minh City, Vietnam; 2 Faculty of Information Technology, Nguyen Tat Thanh University, Ho Chi Minh City, Vietnam; Nanjing University of Aeronautics and Astronautics, CHINA

## Abstract

In recent years, graph neural networks (GNNs) have emerged as a dominant paradigm for learning from graph-structured data, achieving strong performance across a wide range of graph mining tasks, including node classification, clustering, and relational inference. Despite these advances, most existing GNN architectures primarily rely on message-passing mechanisms that aggregate smoothed high-order local neighborhood information. While effective for capturing local proximity patterns, such designs often struggle to model richer multi-level graph structures, particularly in scenarios where both fine-grained local interactions and holistic global organization are essential. From a structural perspective, graph representation learning inherently involves information distributed across multiple levels. Local proximity cues, such as node degrees and common neighbors, capture immediate relational patterns, whereas global structural characteristics, including long-range dependencies and overall graph topology, provide complementary high-order information. However, conventional GNN models typically entangle these heterogeneous signals within a unified aggregation process, leading to suboptimal representations. To address this limitation, we propose DAGRL, a dual attention-based graph representation learning framework that explicitly disentangles and integrates multi-level structural information. The proposed model employs dedicated graph neural components to learn structure-specific representations and utilizes a dual attention mechanism to adaptively fuse them into a unified embedding space. Extensive experiments demonstrate that DAGRL consistently outperforms existing methods across multiple benchmark datasets.

## 1. Introduction

For many years, link prediction [1–3] has been recognized as a fundamental research problem with wide-ranging applications across multiple domains [4,5], including social network analysis, knowledge graph completion, recommender systems, and bioinformatics. In general, link prediction aims to identify missing or potential relationships between pairs of nodes in each graph. Numerous real-world problems can be naturally formulated under this paradigm. For instance, in social networking platforms such as Facebook or Twitter, tasks like friendship suggestion and group recommendation can be modeled as predicting links between users or between users and communities. Similarly, in knowledge graphs such as YAGO and Freebase, link prediction is commonly used to infer missing relationships between entities, also known as knowledge graph completion. Moreover, recent studies in wireless and dynamic network systems show that learning-based models can effectively capture evolving connectivity patterns and nonlinear interactions among entities [6]. These problems can be naturally interpreted as link prediction tasks, where the goal is to estimate the likelihood of relationships based on structural and contextual features [7]. Traditionally, link prediction has been addressed using heuristic or hand-crafted feature engineering methods combined with classical machine learning algorithms. These approaches rely on predefined structural features, such as node degree, common neighbors, Katz index, preferential attachment, and shortest path distance, to characterize node relationships. However, such topology-based methods often suffer from limited generalization capability and struggle to adapt to diverse graph structures. To overcome these limitations, node representation learning [8] or graph embedding, has emerged as a dominant paradigm. Techniques such as DeepWalk [9], LINE [10], Node2Vec [11] learn low-dimensional vector representations of nodes that capture local structural properties. Despite their effectiveness, these methods are inherently transductive and primarily focus on local proximity, limiting their ability to generalize to unseen nodes and capture higher-order, multi-level graph structures. Consequently, there remains a need for more advanced graph learning frameworks capable of modeling both local and global structural information for improved link prediction performance.

### 1.1. Graph neural networks for link prediction: evolution and progress

With the rapid advancement of deep learning (DL), GNN [3] have emerged as one of the most powerful and widely adopted neural architectures for learning from graph-structured data. Within the field of network representation learning, GNN-based models have been developed to overcome the limitations of traditional network embedding techniques, particularly in preserving high-order structural information and enabling inductive, task-driven learning. As a result, a variety of GNN architectures, such as: GCN [12], GraphSAGE [13], APPNP [14], GAT [15], GIN [16], GRAND [17], DAGNN [18], and GATv2 [19], have demonstrated significant improvements across a range of graph learning tasks through end-to-end optimization of node and graph representations. These models typically learn node representations by jointly leveraging graph topology and node attributes through message-passing mechanisms. In the context of link prediction, the learned node embeddings are used to estimate the likelihood of relationships between node pairs based on their structural similarity in the latent space. Consequently, GNN-based approaches have become a dominant framework for modeling complex relational patterns in graphs. Existing GNN-based link prediction methods can generally be categorized into two paradigms: node-based and subgraph-based approaches. Node-based methods, such as GCN [12], and GAE/VGAE [20], learn link representations directly from node embeddings obtained via neighborhood aggregation. In contrast, subgraph-based methods, such as SEAL [21], DE-GNN [22], focus on learning representations from localized subgraphs extracted around target node pairs. Despite their effectiveness, both paradigms primarily emphasize high-order local structural information. However, recent studies have highlighted that conventional GNN architectures are inherently limited in capturing global structural dependencies. Due to the reliance on localized message passing, information propagation is restricted to k-hop neighborhoods, even when stacking multiple layers. Increasing network depth to capture long-range dependencies often leads to over-smoothing and over-squashing issues [23], which degrade representation quality and hinder learning. Therefore, it is crucial to develop more advanced graph learning mechanisms that can effectively integrate both local and global structural information. Such multi-level graph representation learning is considered as essential for improving the performance of fundamental graph mining tasks, including link prediction.

### 1.2. Motivations and contributions

**Fig 1:**
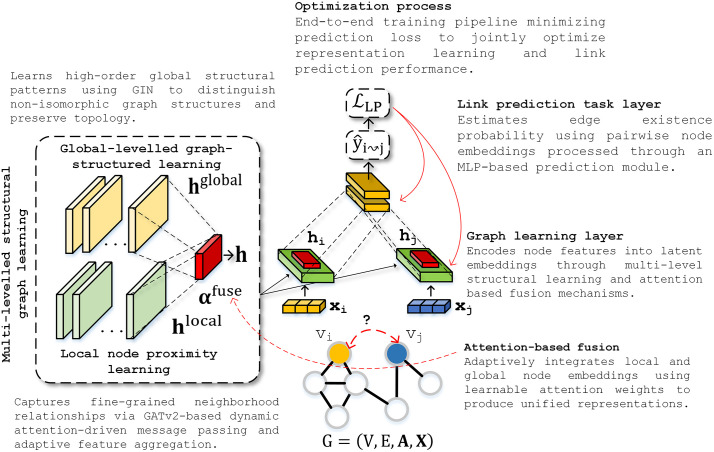
Illustration of the overall architecture, neural components, and end-to-end learning workflow of our proposed DAGRL model for multi-level graph representation learning and link prediction tasks.

Motivated by recent advances in graph neural architectures for link prediction, we propose a novel multi-view graph representation learning framework, namely DAGRL. Fig 1 illustrates the overall architecture of the proposed model along with its corresponding learning process. In our DAGRL model, a dual-attention mechanism is introduced to effectively capture and integrate both high-order local proximity structures and global graph structural information. Specifically, for local representation learning, we adopt a message-passing strategy based on the dynamic attention mechanism of the GATv2 model [19], which enables adaptive and expressive aggregation of neighborhood features. This design allows the model to selectively focus on the most informative neighboring nodes, thereby enhancing local structural representation. For global structural learning, we incorporate the expressive graph learning paradigm of the GIN [16], where node aggregation and transformation functions are parameterized using flexible neural networks such as MLPs. This component emphasizes the preservation of global graph topology by mapping structurally distinct graphs into discriminative embedding spaces. To effectively unify these complementary representations, we further introduce an attention-based fusion mechanism that adaptively integrates multi-view node embedding into a coherent representation space. All components of DAGRL are trained in an end-to-end manner, with a final MLP-based decoder for link prediction. Overall, the main contributions of this paper can be summarized as follows:

- **Dual-attention multi-levelled graph representation learning framework**. First of all, we propose a novel dual-attention-based multi-view graph representation learning framework, named DAGRL, which explicitly disentangles and jointly models both high-order local proximity and global structural information. Unlike conventional GNN-based approaches that implicitly entangle these heterogeneous signals within a unified aggregation process, our framework introduces dedicated learning components for each structural view. This design enables a more principled and interpretable representation learning process, leading to richer and more expressive node embeddings that effectively capture complex graph semantics.
- **Adaptive attention-based multi- levelled embedding fusion mechanism**. Secondly, we develop an attention-driven fusion mechanism to adaptively integrate multi-view representations learned from different graph neural components into a unified embedding space. Inspired by node-level attention mechanisms, this fusion strategy dynamically assigns importance weights to each structural view, rather than relying on simple concatenation or averaging. As a result, the model can selectively emphasize the most informative features from both local and global perspectives, significantly enhancing the quality of learned representations.
- **End-to-end task-driven learning architecture for link prediction**. Thirdly, we design an end-to-end learning framework that seamlessly integrates multi-view representation learning with task-specific optimization through an MLP-based prediction module. This unified architecture ensures that the learned node embeddings are directly optimized for the link prediction objective, improving both learning efficiency and predictive performance compared to loosely coupled or multi-stage approaches.
- **Comprehensive empirical validation and ablation analysis**. Finally, we conduct extensive experiments and ablation studies on multiple real-world benchmark datasets with diverse structural characteristics. The results demonstrate that our introduced DAGRL model consistently outperforms both classical and state-of-the-art GNN baselines. Furthermore, the ablation analysis validates the effectiveness of each component and highlights the critical role of integrating local and global structural information through the proposed dual-attention mechanism.

**Novel contributions and distinction of our work from existing graph neural architectures**. Although DAGRL incorporates components inspired by existing GNN-based architectures, such as GATv2, GIN, and attention-based fusion mechanisms, its contribution extends beyond a mere integration of these techniques. In contrast to conventional GNN-based models, which typically entangle local and global structural information within a unified message-passing framework, DAGRL introduces a principled multi-levelled representation learning paradigm that explicitly disentangles and models heterogeneous structural signals through dedicated neural components. Specifically, the local component employs dynamic attention mechanisms to capture fine-grained neighborhood interactions, whereas the global component adopts an expressive aggregation scheme to preserve high-order structural characteristics. Furthermore, DAGRL proposes a novel attention-guided fusion mechanism that adaptively integrates these complementary representations into a unified embedding space, enabling dynamic balancing of multi-level structural information. This design fundamentally differs from prior approaches that rely on implicit aggregation or static combination strategies. In addition, the entire framework is optimized in an end-to-end manner for the link prediction task, ensuring that the learned representations are both structurally informative and task specific. Consequently, the novelty of DAGRL resides in its explicit structural disentanglement, adaptive multi-view integration, as well as unified optimization framework, which collectively distinguish it from existing GNN-based methods

The remainder of this paper is organized into four sections. In the next section, we provide a concise review of recent studies related to graph representation learning and link prediction. Section 3 presents the proposed methodology, including the detailed design and formulation of the DAGRL framework for multi-view graph representation learning and link prediction. Section 4 reports extensive empirical evaluations to demonstrate the effectiveness and superiority of the proposed model. Finally, the last section concludes the paper by summarizing the main findings and discussing potential directions for future research.

## 2. Related works

Within the graph-based/networked data analysis and mining domain, link prediction [1–3] is considered as an important/primitive problem which can be formulated and applied for multiple real-world applications [4,5]. In general, a link prediction model is designed to learn from the historical or current structure of a graph in order to estimate the likelihood of missing or potential links between pairs of nodes. Within graph-based datasets, the link prediction task can be formulated across various application domains, such as product, friendship, or authorship recommendation (e.g., social networks, e-commerce platforms, and bibliographic networks), as well as relationship completion in knowledge graphs and biological networks. Traditionally, link prediction has been formulated as a classification problem and addressed using hand-crafted or heuristic feature engineering approaches, including measures such as node degree, common neighbors, Adamic–Adar index, Katz index, preferential attachment, and shortest path distance. However, these heuristic-based methods suffer from limited generalization capability, as they rely on predefined structural features that are often tailored to specific graph types or tasks. Furthermore, dependence on fixed feature representations restricts their ability to adapt to diverse and complex graph structures, thereby limiting their effectiveness in real-world link prediction scenarios. To overcome these limitations, there are multiple studies that have employed the latent feature representation learning paradigm of NLP’s domain into the graph feature engineering process. There are notable node embedding [8] methods have been proposed at that time, such as: DeepWalk [9], LINE [10], Node2Vec [11], etc. to support for extracting the local proximity structural features from graph’s nodes. Then, these latent feature representations are utilized to facilitate the process of node similarity evaluation to identify the likelihood of link occurrence between them. However, as previous node embedding techniques are considered as too shallow to be able to preserve complex/high-ordered structural information of the given graph. Thus, they still suffered several limitations related to the capability of dealing with link prediction problems within complex graph-structured datasets. Moreover, as network embedding approach is also known as transductive neural learning approach, thus it can neither produce the representations of unseen nodes nor predict links between them.

In recent years, the emergence of GNN [3] in graph-based data analysis and mining domain has shed light for promising enhancements within the link prediction area. In fact, there are several recent studies that have demonstrated the power of GNN-based architecture in coping with complex link prediction problems in both homogeneous/heterogeneous graph-structured datasets. Developing upon graph neural learning framework, most of recent proposed GNN-based link predictive techniques can be categorized into two main approaches which are the node-levelled and subgraph-levelled approaches. Within the node-levelled approach, similar to the employment of node embedding method, the traditional GNN-based architectures, such as: GCN [12], GAE/VGAE [20], GraphSAGE [13], GAT [15], etc. have been employed to estimate the link likelihood occurrence between pairwise nodes by evaluating their corresponding graph-structural feature representations. However, GNN-based architectures are considered an inductive approach which can learn the representations of unseen nodes. Therefore, the GNN-based techniques can be flexibly applied for the link prediction problem in context of graph’s structure independence as well as richer-structural/high-ordered information preserving. On the other side, within the subgraph-based approach [21,22], the graph structural feature embedding and prediction learning processes have been restricted to separated subgraph-levelled structures. Such as within the well-known SEAL [21] model, Zhang, M. et al. proposed approach of extracting the enclosing subgraphs for different target links. Then, the canonical GNN-based models are applied to learn the representations of these subgraphs for dealing with the link prediction problem. However, the previous techniques in both node-based/subgraph-based approaches still mainly concentrate on extracting the high-ordered local proximity information between pairwise nodes to facilitate the relational structural forecasting process. Therefore, they might still suffer several challenges while dealing with complex link prediction problems in which global graph-structured information is required. As a result, there is still room for further studies as well as novel graph learning mechanism proposals in this research direction.

## 3. Methodology and implementation

In this section, we present the overall methodology of the proposed DAGRL model, along with detailed descriptions of its neural components and implementation. In general, our DAGRL model is designed as a dual-attention-assisted graph representation learning framework designed to preserve multi-level structural information from the input graph. The resulting node representations capture both local and global structural characteristics, making them well-suited for a variety of graph learning tasks, particularly link prediction through task-specific fine-tuning. For completeness, we first introduce the formulation of the link prediction problem and briefly review the key background concepts relevant to the proposed DAGRL framework.

### 3.1. Problem formulation and preliminaries

Given a graph structure denoted as: G=(V,E,𝐀,𝐗), where V is the set of n nodes and E⊆V×V is the set of m edges, we consider a general formulation that can accommodate both directed or undirected as well as weighted or unweighted graphs. The adjacency matrix, as: $𝐀\in R^{\text{n}\times \text{n}}$, encodes pairwise relationships between nodes, where each entry: (${\text{a}}_{\text{ij}}$) represents the connection from node (${\text{v}}_{\text{i}}$) to node (${\text{v}}_{\text{j}}$). Specifically, ${\text{a}}_{\text{ij}}=\text{1}$ for unweighted graphs, or ${\text{a}}_{\text{ij}}\in \text{R}$ for weighted graphs, if an edge exists; otherwise, ${\text{a}}_{\text{ij}}=\text{0}$. In directed graphs, ${\text{a}}_{\text{ij}}\neq {\text{a}}_{\text{ji}}$, whereas in undirected graphs, as: (**A**) is symmetric. The node feature matrix: $𝐗\in R^{\text{n}\times {\text{d}}_{\text{feat}}}$ contains the initial feature vectors of all nodes, where each node ${\text{v}}_{\text{i}}\in \text{V}$ is associated with a feature vector, as: ${𝐱}_{\text{i}}\in R^{{\text{d}}_{\text{feat}}}$. The link prediction task aims to learn a mapping function ${\text{f}}_{\text{LP}}$ that estimates the likelihood of a link between node pairs. Formally, the model predicts the existence of missing or potential edges in the complement set: $\hat{\text{E}}=(\text{V}\times \text{V})\setminus \text{E}$. We denote: ${\text{y}}_{\text{i}\to \text{j}}$ and ${\hat{\text{y}}}_{\text{i}\to \text{j}}$ as the ground-truth label and the predicted probability of a link between nodes (${\text{v}}_{\text{i}}$) and (${\text{v}}_{\text{j}}$), respectively. In our implementation, unless otherwise specified, graphs are processed as directed and unweighted, where edge directions are preserved and edge weights are not explicitly utilized during training and evaluation.


$$\begin{array}{c} {\text{f}}_{\text{LP}}:\text{V}\times \text{V}\to 𝐑 \end{array}$$ (1)

$$\begin{array}{c} \overset{\left[\text{l}\right]}{\underset{\text{i}}{𝐡}}=\sigma \left(\overset{\left[\text{l}\right]}{\underset{\text{GNN},\theta }{\text{f}}}\left(\overset{\left[\text{l}-\text{1}\right]}{\underset{\text{i}}{𝐡}},\overset{\left[\text{l}\right]}{\underset{\text{GNN},\text{AGG}}{\text{f}}}\left(\overset{\left[\text{l}-\text{1}\right]}{\underset{\text{j}}{𝐡}}:{\text{v}}_{\text{j}}\;\in 𝒩(\text{i})\right)\right)\right) \end{array}$$ (2)

**Graph neural networks for graph representation learning.** GNNs are widely recognized as state-of-the-art neural architectures for learning representations from graph-structured data in a task-driven manner. Most well-known GNN models are developed based on the message-passing or neighborhood aggregation paradigm, in which the representation of a target (ego) node is iteratively updated by aggregating information from its neighboring nodes. Formally, the message-passing process at each GNN layer can be decomposed into two key operations: feature aggregation, denoted as: ${\text{f}}_{\text{AGG}}$, and feature update, denoted as ${\text{f}}_{\theta }$. At the (l)-th layer, the representation of node (${\text{v}}_{\text{i}}$) is computed by aggregating the representations of its neighbors N(i), followed by a transformation through a learnable function and a nonlinear activation. The general propagation process is described in Equation (2). Here, N(i) denotes the set of neighboring nodes of (${\text{v}}_{\text{i}}$), and (σ) represents the activation function, typically implemented as ReLU [12] or LeakyReLU [15]. Different GNN architectures adopt different aggregation strategies. For instance, the GCN model employs normalized mean aggregation, while GraphSAGE utilizes alternative schemes such as max pooling or mean aggregation. These variations enable GNN-based architectures to effectively capture diverse structural patterns and improve representation learning across different types of graph data.

**Self-attention mechanism in GNN.** In recent years, attention mechanisms [24] have emerged as one of the most influential components in advanced deep neural architectures. In the natural language processing (NLP) domain, multi-headed self-attention, as employed in transformer-based models, enables neural networks to effectively capture contextual dependencies among tokens within input sequences. This mechanism allows models to dynamically focus on the most relevant elements, thereby improving representation quality. Similarly, in the graph neural learning domain, self-attention mechanisms have been widely adopted in models such as GAT [15], GATv2 [19] to enhance the feature aggregation process between nodes. By assigning adaptive importance weights to neighboring nodes, the attention mechanism allows the model to selectively emphasize more informative structural relationships during message passing. As a result, it facilitates the learning of higher-quality, task-oriented node representations. In general, the attention mechanism computes an attention coefficient (${𝐳}_{\text{i↝j}}$) for specific edge: (${\text{e}}_{\text{i↝j}}$) (between ${\text{v}}_{\text{i}}$ and ${\text{v}}_{\text{j}}$ nodes). This attention score reflects the relative importance of node (${\text{v}}_{\text{j}}$) to node (${\text{v}}_{\text{i}}$) during feature aggregation and is typically formulated as shown in Equation (3).


$$\begin{array}{c} {𝐳}_{\text{i↝j}}=\sigma \left({𝐚}^{\text{T}}\left[{\text{f}}_{\text{GNN},\theta }\left({𝐡}_{\text{i}}\right)||{\text{f}}_{\text{GNN},\theta }\left({𝐡}_{\text{j}}\right)\right]\right) \end{array}$$ (3)

$$\begin{array}{c} \alpha_{\text{i↝j}}=\text{softmax}\left({𝐳}_{\text{i↝j}}\right)=\frac{\text{exp}\left({𝐳}_{\text{i↝j}}\right)}{\sum_{{\text{j}}^{\prime }\;\in 𝒩(\text{i})}\text{exp}\left({𝐳}_{\text{i↝}{\text{j}}^{\prime }}\right)} \end{array}$$ (4)

In the Equation (3), the (**a**) presents for the attention-based learnable parameter. Based on the original implementation of GAT model [15], the (σ) activation function in this equation is normally set up as the LeakyReLU function. The ([.||.]) operator presents the vector-based concatenation operation. Then, the computed weighting attention scores are normalized across all the neighborhood nodes of the given (${\text{v}}_{\text{i}}$) and (${\text{v}}_{\text{j}}$) nodes by applying the softmax (as illustrated within the Equation 4).

### 3.2. DAGRL: A dual attention-driven multi-levelled graph learning framework

#### 3.2.1. Link prediction under the multi-levelled graph neural learning paradigm.

Within the proposed DAGRL framework, our primary objective is to enhance link prediction performance by effectively incorporating rich structural information from multiple graph views into the representation learning process. To achieve this, we explicitly decompose the graph embedding module into distinct neural components, each tailored to capture specific structural characteristics. For modeling high-order local proximity relationships between node pairs, we adopt an attention-based message-passing architecture derived from the GAT paradigm [15]. However, to improve the expressiveness and flexibility of attention-based aggregation under dynamic neighborhood interactions, we employ the enhanced variant, namely GATv2 [19]. Compared to the original GAT, GATv2 introduces a dynamic attention formulation that allows the attention coefficients to depend more adaptively on node features, thereby enabling a more expressive and context-aware aggregation mechanism. Concretely, for each edge, the node feature update process in GATv2 [19] is performed by first computing a joint representation through the concatenation of transformed features of the target node (${\text{v}}_{\text{i}}$) and its neighboring node (${\text{v}}_{\text{j}}$). This concatenated representation is then passed through a nonlinear activation function, followed by projection with a learnable attention vector to produce the unnormalized attention coefficient. This formulation contrasts with the static attention mechanism in GAT and enables the model to dynamically adjust the importance of neighboring nodes based on feature interactions. Based on these computed attention scores, neighborhood aggregation is performed to update node representations, where contributions from different neighbors are weighted according to their normalized attention coefficients. Accordingly, the dynamic attention mechanism applied in the high-order local proximity learning component, together with the corresponding node update process for a given edge $\left({\text{e}}_{\text{i↝j}}\right)$, can be formally expressed as follows (see Equation 5):


$$\begin{array}{c} {𝐳}_{\text{i↝j}}={𝐚}^{\text{T}}\text{LeakyReLU}\left({\text{f}}_{\text{GNN},\theta }\left(\left[\overset{\text{local}}{\underset{\text{i}}{𝐡}}||\overset{\text{local}}{\underset{\text{j}}{𝐡}}\right]\right)\right) \end{array}$$

$$\begin{array}{c} \overset{\text{local}}{\underset{\text{i↝j}}{\alpha }}=\text{softmax}\left({𝐳}_{\text{i↝j}}\right) \end{array}$$ (5)

$$\begin{array}{c} \overset{\text{local}}{\underset{\text{i}}{𝐡}}=\text{ReLU}\left(\sum {\mathrm{\backslash nolimits}}_{\text{j}\in N(\text{i})}\overset{\text{local}}{\underset{\text{i↝j}}{\alpha }}{\text{f}}_{\text{GNN},\theta }\left(\overset{\text{local}}{\underset{\text{j}}{𝐡}}\right)\right) \end{array}$$ (6)

$$\begin{array}{c} \overset{\text{global}}{\underset{\text{i}}{𝐡}}=\text{MLP}\left(\left(\text{1}+\varepsilon \right)\overset{\text{global}}{\underset{\text{i}}{𝐡}}+\sum {\mathrm{\backslash nolimits}}_{\text{j}\in N(\text{i})}\overset{\text{global}}{\underset{\text{j}}{𝐡}}\right) \end{array}$$ (7)

Empirical evidence from the original study [19] demonstrates that this modification enhances the expressiveness of the self-attention mechanism, enabling more adaptive and context-aware aggregation of neighborhood information. As a result, the learned node representations become more discriminative by dynamically emphasizing the most relevant neighboring nodes during message passing. Based on the normalized attention coefficients, as: $\left(\overset{\text{local}}{\underset{\text{i↝j}}{\alpha }}\right)$, the local proximity-based representation of each node *i* is computed through weighted aggregation over its neighborhood, yielding the embedding, as: ($\overset{\text{local}}{\underset{\text{i}}{𝐡}}$), as formalized in Equation (6). To complement local structural learning, we further incorporate a global graph-structural representation component based on a high-expressive graph learning paradigm. Specifically, we employ the GIN model [16], which is theoretically grounded in the Weisfeiler–Lehman (WL) graph isomorphism test. This design enables the model to effectively distinguish structurally different (non-isomorphic) graphs by mapping them into distinct embedding spaces. Consequently, the GIN-based component is capable of preserving global topological characteristics and capturing high-order structural patterns during the propagation process, thereby providing complementary information to the local attention-based representations. Within this approach, the updating/aggregation node feature process can be parameterized as (γ) and (φ) mapping functions, as: $\text{GIN}\left(\text{c},𝐗\right)=\varphi \left((\text{1}+\varepsilon ).\gamma (\text{c})+\sum_{\text{x}\in X}\gamma (\text{x})\right)$, for each pair (c,*x*) and we have: c∈𝐗 and: X⊂𝐗 is a multiset of bounded size [16]. Relying on the universal approximation theorem, the (γ) and (φ) mapping functions can be then modelled as the MLP-based architecture to produce the global-structural node representation, represented as: ($\overset{\text{global}}{\underset{\text{i}}{𝐡}}$) (as illustrated in Equation 5). In this equation, the (ε) presents for the trainable parameter.

**Handling of edge weights in graph learning**. It is important to clarify that, although the general graph formulation supports both weighted and unweighted edges, our proposed DAGRL framework does not explicitly utilize edge weights during the learning process. In particular, for graphs that contain weighted relationships, these weights are not incorporated into the attention coefficients or aggregation functions of either the local attention-based component or the global aggregation component. Instead, all edges are treated uniformly based solely on their existence, resulting in a binary connectivity representation during message passing and feature aggregation. This design choice ensures that the learned node representations focus on structural connectivity patterns rather than being influenced by dataset-specific edge weighting schemes, thereby maintaining a unified and generalizable framework across different types of graphs. Nonetheless, incorporating edge weights—such as integrating them into attention mechanisms or aggregation operations—could further enhance the model’s ability to capture fine-grained relational strengths, which we consider as a promising direction for future work.

#### 3.2.2. Self-attention-aided graph embedding fusion & link prediction fine-tuning.

From the generated multi-viewed node representations which are produced by different graph neural architectures, we apply a custom embedding fusion mechanism to produce the final node embeddings, denoted as: (${𝐡}_{\text{i}}$). To do this, we apply a self-attention mechanism which is mainly adopted from previous works in order to intrinsically achieve the proper alignments between node latent features that carry different graph-structural information. The multi-view node embedding fusion process is generally formulated as shown in the Equation (8).


$$\begin{array}{c} \overset{\text{com}}{\underset{\text{i}}{𝐡}}=\left[\overset{\text{local}}{\underset{\text{i}}{𝐡}}||\overset{\text{global}}{\underset{\text{i}}{𝐡}}\right] \end{array}$$

$$\begin{array}{c} \overset{\text{fuse}}{\underset{\text{i}}{\alpha }}=\text{softmax}\left({𝐚}^{\text{fuse}}\overset{\text{com}}{\underset{\text{i}}{𝐡}}\right) \end{array}$$

$$\begin{array}{c} {𝐡}_{\text{i}}=\overset{\text{com}}{\underset{\text{i}}{𝐡}}+\overset{\text{fuse}}{\underset{\text{i}}{\alpha }}\overset{\text{com}}{\underset{\text{i}}{𝐡}} \end{array}$$ (8)

$$\begin{array}{c} {\hat{\text{y}}}_{\text{i↝j}}=\text{MLP}\left({𝐡}_{\text{i}},{𝐡}_{\text{j}}\right) \end{array}$$ (9)

$$\begin{array}{c} {ℒ}_{\text{LP}}=-\sum_{{\text{e}}_{\text{i}⇝\text{j}}\in 𝒯}\left({\text{y}}_{\text{i}⇝\text{j}}\log\left({\hat{y}}_{\text{i}⇝\text{j}}\right)+\left(1-{\text{y}}_{\text{i}⇝\text{j}}\right)\log\left(1-{\hat{y}}_{\text{i}⇝\text{j}}\right)\right) \end{array}$$ (10)

In this equation, the (${𝐚}^{\text{fuse}}\in R^{\text{2d}\times \text{d}}$) present for the trainable weighting parameter of the given embedding fusing component. Within our implementation, at the model initialization state, we set its initial value by using the Xavier uniform distribution method [25]. For link prediction, the proposed model estimates the likelihood of edge existence between a pair of nodes by utilizing their learned representations. Specifically, the final node embeddings are first combined through a concatenation operation, which preserves the complete feature information from both nodes. This combined representation is then fed into an MLP-based architecture that serves as a task-driven prediction layer, enabling the model to capture complex nonlinear relationships between node pairs. The MLP-based decoder transforms the pairwise representation into a scalar score that reflects the probability of link occurrence. By adopting this design, the model can effectively learn discriminative patterns for distinguishing existing and non-existing links. Following standard practices in GNN-based link prediction, we employ the cross-entropy loss function (as defined in Equation 10) to optimize all model parameters in an end-to-end manner with respect to the link prediction objective ($L_{\text{LP}}$). This training strategy ensures that both the representation learning components and the prediction layer are jointly optimized for improved performance.

**Distinction between dual-attention mechanisms.** It is important to emphasize that the proposed DAGRL framework incorporates two distinct attention mechanisms with clearly different roles and operational levels. The first is a local node-level attention mechanism, implemented based on GATv2, which operates at the edge level to adaptively assign importance weights to neighboring nodes during message passing. The second is a view-level fusion attention mechanism, which operates at the representation level to integrate local and global structural embeddings into a unified feature space. Unlike the local attention mechanism that focuses on modeling neighborhood importance, the fusion attention mechanism is designed to dynamically balance multi-level structural information across different views. These two attention mechanisms are complementary and function at different stages of the learning pipeline, contributing jointly to the overall expressiveness of the proposed model.

#### 3.2.3. Computational complexity analysis of our DAGRL model.

To further assess the efficiency and scalability of the proposed DAGRL framework, we analyze its computational complexity with respect to the number of nodes n=|V|, edges m=|E|, and embedding dimension d. The overall computational cost is primarily governed by three components: the local-level learning module, the global-level learning module, and the attention-based fusion mechanism. For the local attention-based component, the computational cost mainly arises from message passing and attention computation over edges, which scales approximately as: O(m·d). The global component similarly performs neighborhood aggregation followed by feature transformations, resulting in a complexity that depends on both edge interactions and node-wise transformations, typically approximated as: $O(\text{m}\cdot \text{d}+\text{n}\cdot {\text{d}}^{\text{2}})$. The fusion module operates at the node level and introduces relatively low overhead, with complexity proportional to: O(n·d), as it combines multi-leveled representations through attention mechanisms. Overall, the computational complexity of DAGRL can be summarized as: $O(\text{m}\cdot \text{d}+\text{n}\cdot {\text{d}}^{\text{2}}),$ which scales linearly with the number of edges and is comparable to existing GNN-based architectures.

## 4. Empirical studies and discussions

In this section, we present comprehensive empirical studies to evaluate the effectiveness of the proposed DAGRL model in graph representation learning and link prediction tasks. We conduct extensive comparative analyses against a range of state-of-the-art graph learning methods to demonstrate the superiority of our approach on real-world benchmark datasets. Furthermore, we provide detailed descriptions of the datasets, experimental settings, and model configurations to ensure clarity and reproducibility of the reported results.

### 4.1. Dataset descriptions

To evaluate the performance of our DAGRL model as well as other comparative baselines (later described within the sub-section 4.4.1), we mainly used 4 graph-based datasets which are available in the well-known Network Repository (NR) online data resource [26]. These datasets are considered as the benchmark/real-world social network based datasets, which are:

- **soc-advogato**: is a well-established social network platform for open-source software collaboration and knowledge exchange among users. This dataset is modeled as a weighted graph, where edges represent user–user relationships associated with trust scores that reflect the strength of interactions. The released version of this dataset contains approximately 6.5K nodes and 53.3K edges, capturing rich relational patterns within a trust-aware social network.
- **soc-hamsterster**: is a web-based social network that captures friendship and family relationships among users. Like the soc-advogato dataset, it is represented as a graph structure where nodes correspond to users and edges denote social connections. The released version of this dataset contains approximately 2.4K nodes and 16.6K user–user relationships.
- **soc-wiki-elec**: is the largest network dataset used in our experiments. It captures election and voting relationships among administrator candidates within the Wikipedia platform. The dataset is constructed from historical voting records derived from Wikipedia page edits, reflecting community-driven decision processes. The released version contains approximately 7.1K nodes and 107K edges, representing a large-scale and structurally complex social network.
- **web-polblogs**: is a well-known political blog network dataset which captures hyperlink relationships between blogs discussing U.S. politics [27]. This dataset is considered as an unweighted graph-based structure where nodes represent blogs and edges denote hyperlinks between them, reflecting information diffusion and community interactions. The released version of this dataset contains about 2.3K edges, with a relatively higher graph density compared to other datasets used in this study. The average node degree is approximately 7.10, indicating a moderately connected but highly polarized network structure, which makes it suitable for evaluating link prediction performance under sparse yet community-driven graph settings.

Overall, the selected datasets cover diverse types of graph structures, including weighted trust networks (soc-advogato), friendship-based social graphs (soc-hamsterster), voting/election networks (soc-wiki-elec), and hyperlink-driven political interaction graphs (web-polblogs). These datasets also vary significantly in scale, density, and structural characteristics, ranging from sparse to relatively dense connectivity patterns. Such diversity enables a more comprehensive evaluation of the proposed DAGRL model under different graph learning scenarios.

### 4.2. Pre-processing procedures, node feature generation, and dataset partitioning strategy

From the original data structures of the aforementioned datasets, we first parse the relationships between nodes and construct graph-based representations tailored for the link prediction task. Specifically, the raw edge information is converted into graph structures, from which both positive and negative edge sets are generated for training and evaluation, following common practices in prior studies [21,22].

**Initial node feature generation through Node2Vec**. To initialize node representations, we employ the Node2Vec model [11] to learn low-dimensional embeddings that capture local structural properties of the graph. The dimensionality of node features is set to ${\text{d}}^{\text{feat}}=\text{6}\text{4}$. For the Node2Vec training process, we configure the number of random walks per node as 5, the walk length as 5, and the number of training iterations as: 5. These configurations ensure a balance between computational efficiency and representation quality across all datasets. In our current implementation, Node2Vec is trained on the full graph, including both training and testing edges. We acknowledge that this setting may introduce a potential risk of structural information leakage, as the embedding process can implicitly encode connectivity patterns associated with test edges. However, this practice is consistent with several prior works in graph representation learning, where Node2Vec is used as a preprocessing step independent of the downstream task-specific training procedure. Importantly, during the training of the proposed DAGRL model, all message-passing operations are performed strictly on the training graph, where test edges have been removed. Therefore, the learning of task-specific representations and link prediction is not directly influenced by test edge information.

**Negative sampling & train/test split strategies**. To construct negative samples for the link prediction task, we adopt a uniform random sampling strategy over all non-existent node pairs in the graph. Specifically, we first derive the complement adjacency matrix by excluding all observed edges and self-loops. From this candidate space, negative edges are randomly sampled to form the negative edge set. The number of negative samples is set equal to the number of positive edges (i.e., a 1:1 ratio), resulting in a balanced binary classification setting. Importantly, since negative samples are drawn exclusively from node pairs that are not connected in the original graph, this process inherently avoids false negatives, ensuring the correctness of the supervision signals during training and evaluation. For all experiments, we adopt a random edge-level splitting strategy with a ratio of 70% for training and 30% for testing. Specifically, the set of observed edges is randomly permuted, after which 70% of edges are assigned to the training set and the remaining 30% are reserved for testing. To prevent information leakage during message passing, all test edges are removed from the original graph when constructing the training graph. As a result, the GNN-based model only propagates information over the training edges; as a result, ensuring that no structural information from the test set is accessible during training. The link prediction model is then trained using the training graph along with the corresponding positive and negative training edges and evaluated on disjoint test edges. Although this random split does not explicitly enforce connectivity constraints, the inherent structural properties of the benchmark datasets (e.g., sufficient edge density) help maintain meaningful graph connectivity for effective representation learning. Overall, the adopted preprocessing pipeline ensures a fair, leakage-free, and reproducible evaluation setting. The combination of balanced negative sampling, strict separation of training and testing edges, and controlled feature initialization allows for a reliable assessment of the proposed DAGRL model across different graph datasets.

### 4.3. Experimental environment and model configurations

All models, including DAGRL and the comparative baselines, are executed on a system equipped with an NVIDIA L40 GPU (48GB GDDR6), ensuring efficient large-scale graph processing and accelerated training. Regarding model configurations, for the local-level graph representation learning component (as described in sub-section 3.2.1), we set the number of graph attention-based hidden layers ${\text{k}}^{\text{local}}=\text{3}$, with the number of attention heads, as: ${\text{n}}^{\text{head}}=\text{4}$. For the global-level structural learning component implemented using GIN-based architecture, the number of hidden layers is set to: ${\text{k}}^{\text{global}}=\text{3}$. The node embedding dimensionalities for all GNN-based architectures is consistently configured as: ${\text{d}}^{\text{GNN}}=\text{64}$. During training, both the proposed DAGRL model and all baseline methods are trained for a maximum of 200 epochs. The learning rate is fixed at: $\eta =\text{1}\times {\text{10}}^{-\text{4}}$ for all models to ensure a fair comparison. For performance evaluation, we adopt different standard evaluation metrics, such as: F1-Macro, average precision (AP), and area under the ROC curve (AUC) as the primary metric, following common practices in prior link prediction studies. Table 1 summarizes the key architectural and training configurations used in DAGRL, ensuring consistency across local and global components. The unified parameter settings enable fair comparison with baseline models while maintaining stable and efficient learning performance.

**Table 1:** Configuration settings of the proposed DAGRL model for multi-level graph representation learning and link prediction tasks.

Parameter/configuration		Value/setting
**Local component**	Number of layers (${\text{k}}^{\text{local}}$)	3
	Number of attention heads (${\text{n}}^{\text{head}}$)	4
**Global component**	Number of Layers (${\text{k}}^{\text{global}}$)	3
**Initial node embedding**	Node Embedding Dimension (${\text{d}}^{\text{GNN}}$)	64
**Training process**	Number of Epochs	200
	Learning Rate (η)	$\text{1}\times {\text{10}}^{-\text{4}}$

### 4.4. Experimental results and discussions

#### 4.4.1. Implementation of comparative baselines.

For the comparative studies in this paper, we implemented several well-established graph embedding methods designed for both general graph representation learning and link prediction tasks. The considered baselines include: GCN [12], GraphSAGE [13], APPNP [14], GAT [15], GIN [16], GRAND [17], DAGNN [18] and GATv2 [19]. For local neighborhood-based GNN architectures, including GCN, GraphSAGE, GAT, GRAND, and GATv2, we configured the number of hidden layers and node embedding dimensionality to be consistent with our proposed DAGRL model, using three layers and an embedding size of 64. Similarly, for global-structure-aware models such as GIN, we set the number of hidden layers to three and the embedding dimensionality to 64 to ensure fair comparison. To comprehensively evaluate link prediction performance, all baseline models were equipped with an MLP-based decoder, following the same task-driven design as our proposed DAGRL model. Furthermore, all remaining training configurations, including learning rate, optimization strategy, and training epochs, were kept identical across all models to ensure a fair and unbiased comparison.

#### 4.4.2. Performance comparison on link prediction task.

**Fig 2:**
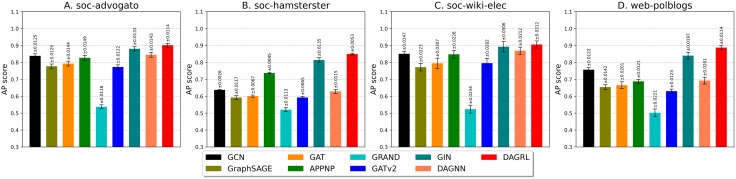
Comparative evaluation of Average Precision (AP) scores across multiple GNN baselines and the proposed DAGRL model on four benchmark social network datasets, highlighting improvements in link prediction performance.

**Fig 3:**
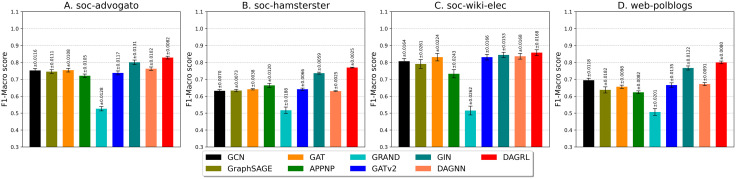
Performance comparison of F1-Macro scores for our DAGRL model and various GNN-based models on four benchmark datasets, demonstrating enhanced classification balance and effectiveness in capturing complex graph structural patterns.

**Fig 4:**
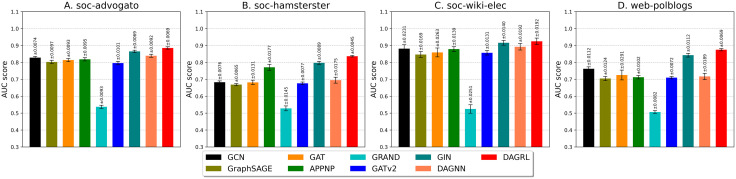
ROC-AUC comparison between our DAGRL model and representative GNN baselines across four social network datasets, illustrating improved discriminative capability and robustness in modeling multi-level graph structural information.

To comprehensively evaluate the effectiveness of the proposed DAGRL framework, we conduct experiments on four benchmark social network datasets, including soc-advogato, soc-hamsterster, soc-wiki-elec, and web-polblogs, using three complementary evaluation metrics: Average Precision (AP) (Fig 2), F1-Macro (Fig 3), and ROC-AUC (Fig 4) with mean ± standard deviation across multiple runs. Overall, our DAGRL model consistently achieves the best performance across all datasets and evaluation metrics, outperforming both classical GNN-based baselines and more advanced state-of-the-art architectures. This demonstrates the effectiveness of the proposed dual-attention mechanism in jointly modeling local proximity and global structural information. On the soc-advogato dataset, our proposed DAGRL model achieves the highest performance across all metrics. Compared with previous GNN baselines, such as: GCN, GraphSAGE, and GAT, our proposed DAGRL model improves AP by 12.44% on average, while outperforming more advanced models such as GATv2 by 16.75%. When compared with global-structure-aware models such as GIN and DAGNN, our introduced DAGRL model still achieves consistent gains of 4.46% on average.

Similar trends are observed in F1-Macro and ROC-AUC, where DAGRL improves over previous GNN baselines by 10.20% and 7.07%, respectively. These results indicate that combining local and global structural information is particularly beneficial in moderately dense and weighted graphs. On the soc-hamsterster dataset, DAGRL demonstrates significantly stronger improvements. Compared with previous GNN baselines, DAGRL improves AP by 39.15% on average, while achieving gains of 42.58% over GATv2.

Compared with more advanced baselines such as GIN and DAGNN, DAGRL still improves performance by 4.55% on average. In terms of F1-Macro and ROC-AUC, DAGRL achieves improvements of 22.25% and 23.52%, respectively, over previous GNN baselines. These results highlight that DAGRL is highly effective in sparse graphs where local message-passing alone is insufficient. Within the soc-wiki-elec dataset, our DAGRL model continues to outperform all baselines across all evaluation metrics. Compared with previous GNN baselines, DAGRL improves AP by 12.01% on average, while achieving an additional 13.01% improvement over GATv2. Compared with stronger baselines such as GIN and DAGNN, DAGRL still provides consistent gains of 1.29%–2.15%. For F1-Macro and ROC-AUC, DAGRL improves over previous GNN baselines by 6.39% and 5.11%, respectively. These results indicate that DAGRL remains effective even in larger and more complex graph structures. On the web-polblogs dataset, our DAGRL model achieves the most significant improvements. Compared with previous GNN baselines, DAGRL improves AP by 28.88% on average, and achieves an improvement of 41.49% over GATv2. Compared with advanced models such as GIN and DAGNN, DAGRL still improves performance by 5.99% on average. For F1-Macro and ROC-AUC, our proposed DAGRL model improves over previous GNN baselines by 14.94% and 15.13%, respectively. These results demonstrate that DAGRL is highly robust in handling sparse and highly polarized graph structures. From a broader perspective, models that rely purely on local neighborhood aggregation generally exhibit lower performance, especially in sparse datasets. In contrast, models that incorporate global structural information achieve better results, confirming the importance of capturing high-order graph dependencies. However, these approaches still lack the ability to dynamically balance local and global representations. Our proposed DAGRL model addresses this limitation by explicitly disentangling local and global learning processes and integrating them through an attention-based fusion mechanism. This design enables the model to adaptively prioritize the most informative structural features, resulting in more expressive node representations.

Consequently, the proposed DAGRL model consistently outperforms both classical and state-of-the-art baselines across all datasets and evaluation metrics. The reported standard deviations further indicate that DAGRL maintains stable and reliable performance across multiple runs. These improvements are consistent across different datasets and metrics, demonstrating the robustness of the proposed approach. Overall, DAGRL achieves superior performance under all experimental settings, with improvements reaching up to 39.15% in AP, 22.25% in F1-Macro, and 23.52% in ROC-AUC over previous GNN baselines. These results strongly validate the effectiveness of the proposed dual-attention multi-view graph representation learning framework.

### 4.5. Model’s stability and parameter sensitivity analysis

In this section, we present additional empirical analyses to comprehensively evaluate the effectiveness, stability, and configuration sensitivity of the proposed DAGRL model in graph representation learning and link prediction tasks. We first conduct ablation studies to investigate the individual contributions of the local-level and global-level graph learning components, as well as their integrated multi-level formulation in DAGRL. By comparing these configurations, we aim to explicitly validate the effectiveness of the proposed dual-attention fusion mechanism. Furthermore, we analyze the stability of the model under different training conditions, including variations in training effort (i.e., number of training epochs) and data availability (i.e., different train/test split ratios). These experiments provide insights into the robustness and learning behavior of DAGRL, as well as its ability to maintain strong performance under different training settings for the link prediction problem.

**Fig 5:**
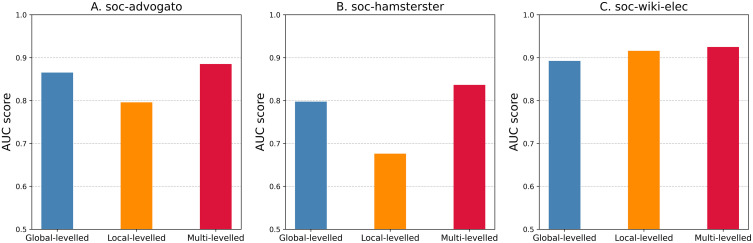
Ablation study comparing global-level, local-level, and multi-level (DAGRL) representations across three benchmark datasets, demonstrating the effectiveness of dual-attention fusion in improving link prediction performance under different structural learning settings.

To assess the contribution of individual components, we conduct an ablation study by evaluating three configurations: local-level learning, global-level learning, and the proposed multi-level DAGRL model. The local-level configuration corresponds to the GATv2-based component, while the global-level configuration is implemented using the GIN-based component. As shown in Fig 5, the multi-level DAGRL model consistently achieves the best performance across all datasets. On the soc-advogato dataset, the global-level configuration already outperforms the local-level one, indicating the importance of capturing higher-order structural dependencies. However, DAGRL further improves over both settings, demonstrating that combining local and global representations leads to more expressive embeddings. A similar trend is observed on the soc-hamsterster dataset, where the performance gap between local and global models becomes more significant due to the sparsity of the graph. In this case, DAGRL provides substantial gains over both configurations, highlighting its ability to compensate for weak local signals through global structural learning. On the soc-wiki-elec dataset, the local-level model achieves competitive performance compared to the global-level model, suggesting that both local and global information are important in large and dense graphs. Nevertheless, DAGRL still achieves the highest performance, confirming that adaptive fusion of multi-level features provides additional benefits beyond individual components. Across all datasets, the multi-level configuration consistently outperforms both single-level baselines, validating the effectiveness of the proposed dual-attention fusion mechanism.

**Fig 6:**
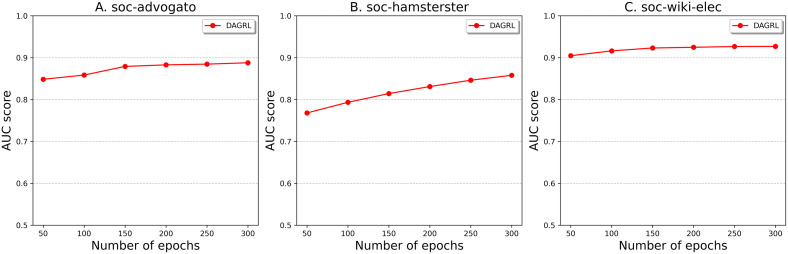
Evaluation of training effort and learning efficiency of the proposed DAGRL model for link prediction across multiple benchmark datasets under varying training configurations.

For the analysis of training epoch configuration, we varied the number of training epochs from 50 to 300 while keeping all other settings consistent with those described in the previous subsection. We then evaluated the corresponding changes in AUC performance of the proposed DAGRL model on the soc-advogato, soc-hamsterster, and soc-wiki-elec datasets. As illustrated in Fig 6, the results indicate that DAGRL achieves stable and high-performance link prediction with a relatively small number of training epochs, converging around 150 epochs for the soc-advogato and soc-wiki-elec datasets, and approximately 200 epochs for the soc-hamsterster dataset.

**Fig 7:**
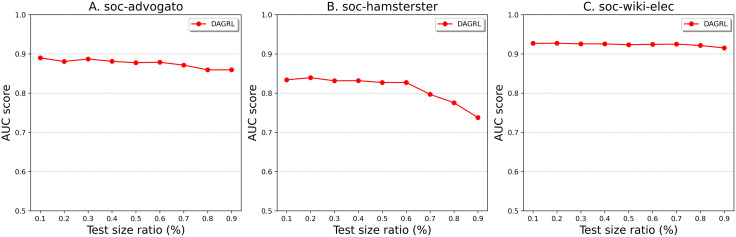
Analysis of the stability of the proposed DAGRL model for link prediction across multiple datasets under varying train/test split ratios and data availability conditions.

**Fig 8:**
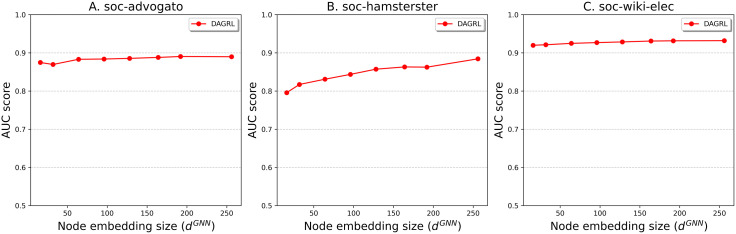
Ablation study on the impact of GNN-based node embedding size ($d^{GNN}$) on link prediction performance across different datasets and graph structural learning settings.

**Fig 9:**
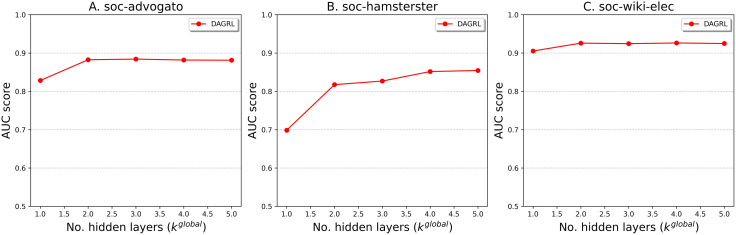
Ablation studies on the influence of GNN-based number of layers ($k^{global}$) parameter for the global graph-structural representation learning component in our DAGRL model.

**Fig 10:**
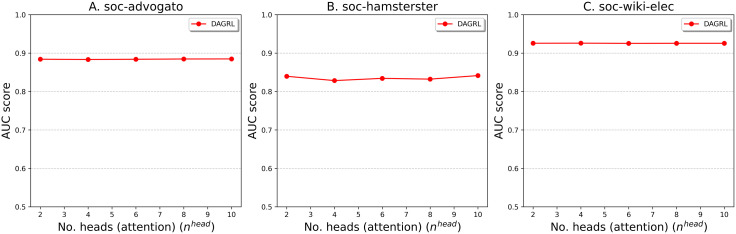
Extensive studies on the configurations of multi-headed attention mechanism ($n^{head}$) for the high-ordered node proximity representation learning component in our DAGRL model.

We further investigate the influence of training data availability on the performance of the proposed DAGRL model in both graph representation learning and link prediction tasks. To this end, we vary the train/test split ratios, where the test size ranges from 0.1 to 0.9, and evaluate the corresponding changes in model performance. As shown in Fig 7, the results indicate that our DAGRL model can achieve high accuracy with relatively limited training data, particularly on the soc-advogato and soc-wiki-elec datasets. In contrast, for the smaller and sparser soc-hamsterster dataset, the model requires at least 40% of the data for training to obtain stable and reasonable link prediction performance. To further investigate the impact of different graph learning components within the proposed DAGRL model, we conduct a series of experiments analyzing key architectural configurations associated with both local and global structural learning. Specifically, we examine the effects of the global component depth (number of hidden layers ${\text{k}}^{\text{global}}$), the embedding dimensionality: (${\text{d}}^{\text{GNN}}$), and the number of attention heads (${\text{n}}^{\text{head}}$) in the local attention-based module. For the embedding dimensionality (${\text{d}}^{\text{GNN}}$), we vary its value within the range of 16–256 and evaluate the corresponding AUC performance across all datasets. As shown in Fig 8, the results indicate that DAGRL is relatively insensitive to this parameter, as performance remains stable across different settings. However, the best performance is consistently achieved when (${\text{d}}^{\text{GNN}}$) is around 200, suggesting this as a suitable configuration for diverse graph datasets. Next, we analyze the effect of the number of hidden layers in the global component by varying (${\text{k}}^{\text{global}}$) from 1 to 5. The results, presented in Fig 9, show that increasing the depth generally improves performance, with the model achieving stable and strong results at around 3-layers. This indicates that a moderate depth is sufficient to effectively capture global structural information without introducing unnecessary complexity. Finally, we evaluate the influence of the number of attention heads (${\text{n}}^{\text{head}}$) on the local component, as illustrated in the Fig 10. The results demonstrate that DAGRL is also largely insensitive to this parameter, as variations in the number of attention heads do not lead to significant changes in AUC performance across all datasets.

## 5. Conclusion and future works

In this paper, we present a novel multi-levelled graph representation learning technique which is developed under the integration between multi-modal graph neural learning and dual attention mechanism, called DAGRL. More specifically, within our proposed DAGRL in order to achieve multiple graph-structural view information from the input graph, we incorporate different graph neural networks which are separately designed to capture different structural views of the graph. In our approach, the dynamic graph attention network is mainly utilized to capture and learn the local node proximity-based representations. Then, within the global graph-structural representation learning aspect, we adopted the existing high-expressive graph neural learning approach to capture and learn the difference between the structure-varied input graphs. Then, in order to achieve the unified and multi-viewed feature aligned representations of nodes within the given graph, we employ a custom embedding fusion mechanism which is designed as a self-attention mechanism. This attention-assisted fusion component is considered to be able to softly merge and transform multiple view-varied node representations, that are produced by different GNN-based architectures into unified and task-friendly embedding spaces. Thus, they can be later utilized to facilitate and achieve better performance for the link prediction problem. The extensive empirical studies within several real-world social networked datasets have demonstrated the effectiveness as well as outperformance of our proposed DAGRL model in comparison with recent state-of-the-art GNN-based architectures. In future work, we intend to incorporate more recent advanced graph neural architecture into our proposed DAGRL model which can enable us to capture significant topological features from the input graphs. Thus, it can directly improve not only the quality of learned node representations as well as the performance of multiple task-driven learning objectives in the after all.

## References

[1] Gui C. Link prediction based on spectral analysis. PLoS One. 2024;19(1):e0287385. doi: 10.1371/journal.pone.0287385 38165881 PMC10760775

[2] Kumar A, Singh SS, Singh K, Biswas B. Link prediction techniques, applications, and performance: a survey. Physica A: Stat Mech Appl. 2020;553:124289. doi: 10.1016/j.physa.2020.124289

[3] Wu Z, Pan S, Chen F, Long G, Zhang C, Yu PS. A comprehensive survey on graph neural networks. IEEE Trans Neural Netw Learn Syst. 2021;32(1):4–24. doi: 10.1109/TNNLS.2020.2978386 32217482

[4] Peng S, Yang H, Yamamoto A. BERT4FCA: A method for bipartite link prediction using formal concept analysis and BERT. PLoS One. 2024;19(6):e0304858. doi: 10.1371/journal.pone.0304858 38837990 PMC11152277

[5] Shen Y, Ren Y, Zhang Y. Evolution mechanism of industrial network in Yangtze River Delta region from the perspective of link prediction. PLoS One. 2024;19(9):e0308544. doi: 10.1371/journal.pone.0308544 39302948 PMC11414917

[6] Hassan MA, Imad M, Hassan T, Ullah F, Ahmad S. Impact of Routing Techniques and Mobility Models on Flying Ad Hoc Networks. Studies in Computational Intelligence for Unmanned Aerial Vehicles Communication Networks. Springer International Publishing; 2022. pp. 111–29. doi: 10.1007/978-3-030-97113-7_7

[7] Hassan MA, Granelli F. Harnessing 1D-CNN for received power prediction in sub-6 GHz RIS: part I. In: 2025 IEEE International Conference on Communications Workshops. 2025. doi: 10.1109/ICCWorkshops67674.2025.11162261

[8] Cui P, Wang X, Pei J, Zhu W. A survey on network embedding. IEEE Trans Knowl Data Eng. 2019;31(5):833–52. doi: 10.1109/tkde.2018.2849727

[9] Perozzi B, Al-Rfou R, Skiena S. Deepwalk: Online learning of social representations. In: SIGKDD. 2014. pp. 701–10. doi: 10.1145/2623330.2623732

[10] Tang J, Qu M, Wang M, Zhang M, Yan J, Mei Q. Large-scale information network embedding. In: WWW. 2015. pp. 1067–77. doi: 10.1145/2736277.2741093

[11] Grover A, Leskovec J. Node2vec: Scalable feature learning for networks. In: SIGKDD. 2016. 855–64. doi: 10.1145/2939672.2939754 PMC5108654 27853626

[12] Kipf TN, Welling M. Semi-supervised classification with graph convolutional networks. In: ICLR. 2016. doi: 10.48550/arXiv.1609.02907

[13] Hamilton W, Ying Z, Leskovec J. Inductive representation learning on large graphs. In: NeurIPS. 2017. 1025–35. doi: 10.48550/arXiv.1706.02216

[14] Gasteiger J, Bojchevski A, Günnemann S. Predict then propagate: Graph neural networks meet personalized PageRank. In: ICLR. 2018. doi: 10.48550/arXiv.1810.05997

[15] Veličković P, Cucurull G, Casanova A, Romero A, Lio P, Bengio Y. Graph Attention Networks. In: ICLR. 2018. doi: 10.48550/arXiv.1710.10903

[16] Xu K, Hu W, Leskovec J, Jegelka S. How powerful are graph neural networks? In: ICLR. 2019. doi: 10.48550/arXiv.1810.00826

[17] Feng W, Zhang J, Dong Y, Han Y, Luan H, Xu Q, et al. Graph random neural networks for semi-supervised learning on graphs. In: NeurIPS. 2020. pp. 22092–103. doi: 10.48550/arXiv.2005.11079

[18] Liu M, Gao H, Ji S. Towards deeper graph neural networks. In: SIGKDD. 2020. pp. 338–48. doi: 10.1145/3394486.3403076

[19] Brody S, Alon U, Yahav E. How Attentive are Graph Attention Networks? In: ICLR. 2022. doi: 10.48550/arXiv.2105.14491

[20] Kipf TN, Welling M. Variational graph auto-encoders. arXiv. 2016. doi: 10.48550/arXiv.1611.07308

[21] Zhang M, Chen Y. Link prediction based on graph neural networks. In: NeurIPS. 2018. pp. 5171–81. doi: 10.48550/arXiv.1802.09691

[22] Li P, Wang Y, Wang H, Leskovec J. Distance encoding: Design provably more powerful neural networks for graph representation learning. In: NeurIPS. 2020. pp. 4465–78. doi: 10.48550/arXiv.2009.00142

[23] Topping J, Di Giovanni F, Chamberlain BP, Dong X, Bronstein MM. Understanding over-squashing and bottlenecks on graphs via curvature. In: ICLR. 2022. doi: 10.48550/arXiv.2111.14522

[24] Vaswani A, Shazeer N, Parmar N, Uszkoreit J, Jones L, Gomez AN, et al. Attention is all you need. NeurIPS. 2017. pp. 6000–10. doi: 10.48550/arXiv.1706.03762

[25] Glorot X, Bengio Y. Understanding the difficulty of training deep feedforward neural networks. In: AISTATS. 2010. pp. 249–56. doi: N/A

[26] Rossi R, Ahmed N. The network data repository with interactive graph analytics and visualization. In: AAAI. 2015. pp. 4292–3. doi: 10.1609/aaai.v29i1.9277

[27] Adamic LA, Glance N. The political blogosphere and the 2004 US election: divided they blog. In: Proceedings of the 3rd International Workshop on Link discovery. 2005. pp. 36–43. doi: 10.1145/1134271.1134277

